# Does circumferential casting prevent fracture redisplacement in reduced distal radius fractures? A retrospective multicentre study

**DOI:** 10.1186/s13018-021-02866-9

**Published:** 2021-12-20

**Authors:** A. C. Berger, B. Barvelink, M. Reijman, T. Gosens, G. A. Kraan, M. R. De Vries, M. H. J. Verhofstad, K. W. W. Lansink, P. F. W. Hannemann, J. W. Colaris

**Affiliations:** 1 Department of Orthopedic Surgery, Erasmus MC Rotterdam, University Medical Center, Doctor Molewaterplein 40, 3015 GD Rotterdam, The Netherlands; 2 Department of Orthopedic Surgery, Elisabeth Tweesteden Hospital, Hilvarenbeekse Weg 60, 5022 GC Tilburg, The Netherlands; 3 Department of Orthopedic Surgery, Reinier de Graaf Gasthuis, Reinier de Graafweg 5, 2625 AD Delft, The Netherlands; 4 Department of Surgery, Reinier de Graaf Gasthuis, Reinier de Graafweg 5, 2625 AD Delft, The Netherlands; 5 Trauma Research Unit, Department of Surgery, Erasmus MC, University Medical Center Rotterdam, Doctor Molewaterplein 40, 3015 GD Rotterdam, The Netherlands; 6 Department of Surgery, Elisabeth Tweesteden Hospital, Hilvarenbeekse Weg 60, 5022 GC Tilburg, The Netherlands; 7 Department of Trauma Surgery, Maastricht University Medical Center, P. Debyelaan 25, 6229 HX Maastricht, The Netherlands

**Keywords:** Distal radius fracture, Circumferential cast, Splint, Redisplacement, Fracture, Bone

## Abstract

**Background:**

This study evaluates whether a circumferential cast compared to a plaster splint leads to less fracture redisplacement in reduced extra-articular distal radius fractures (DRFs).

**Methods:**

This retrospective multicentre study was performed in four hospitals (two teaching hospitals and two academic hospitals). Adult patients with a displaced extra-articular DRF, treated with closed reduction, were included. Patients were included from a 5-year period (January 2012–January 2017). According to the hospital protocol, fractures were immobilized with a below elbow circumferential cast (CC) or a plaster splint (PS). The primary outcome concerned the difference in the occurrence of fracture redisplacement at one-week follow-up.

**Results:**

A total of 500 patients were included in this study (PS *n* = 184, CC *n* = 316). At one-week follow-up, fracture redisplacement occurred in 52 patients (17%) treated with a CC compared to 53 patients (29%) treated with a PS. This difference was statistically significant (*p* = 0.001).

**Conclusion:**

This study suggests that treatment of reduced DRFs with a circumferential cast might cause less fracture redisplacement at 1-week follow-up compared to treatment with a plaster splint.

*Level of Evidence* Level III, Retrospective study.

## Introduction

Distal radius fractures (DRFs) are the most common fractures seen in the emergency room. Despite its high incidence, a worldwide diversity in treatment strategies exists. This is especially true for displaced DRFs in the adult population [1, 2].

Displaced DRFs are generally reduced and immobilized in either a plaster splint (PS) or a circumferential cast (CC). Unfortunately, 30–40% of reduced DRFs are unstable which results in fracture redisplacement during the cast immobilization period [3–5]. To prevent fracture redisplacement, the choice for early surgical reduction and plate fixation is gaining popularity [5]. Concerning functional outcome and complication risks, the clinically relevant benefit of surgery in comparison with cast immobilization is not convincing. For a large group of patients, especially elderly people, cast immobilization is therefore still the first choice of treatment [6].

It would be ideal to predict fracture redisplacement of DRFs in an early stage to aid physicians in their decision making whether to perform surgery or not. The scope of many studies in displaced DRFs is focused on defining fracture characteristics predicting fracture instability (e.g. age, degree of initial displacement and metaphyseal comminution of the fracture) [4, 7, 8]. However, good-quality evidence concerning the influence of the type of casting (PS or CC) on fracture redisplacement is lacking.

The choice for a CC or PS is usually based on the hospitals protocol and preference of the treating physician [6, 9]. A potential benefit of circumferential casting is more stability during fracture immobilization [3]. A possible benefit of splinting is the allowance of soft tissue swelling which may reduce pain and the risk of a compartment syndrome [10].

No evidence exists yet that shows superiority of one technique above the other regarding fracture redisplacement in reduced DRFs [9, 11]. This study aims to evaluate whether a circumferential cast compared to a plaster splint reduces the risk of fracture redisplacement in reduced extra-articular distal radius fractures in adults during the first treatment week.

## Methods

This manuscript is written according to the Strengthening the Reporting of Observational Studies in Epidemiology (STROBE) guidelines [12].

### Study design

This retrospective multicentre study was conducted in the Netherlands. Patient selection took place in four hospitals (two teaching hospitals and two academic medical centres). Eligible patients who visited the emergency room of one of the participating hospitals between January 2012 and January 2017 were included.

### Data collection

Adult patients (≥ 18 years) with a displaced extra-articular distal radius fracture (AO/OTA classification 23-A.2 (simple) and 23-A.3 (metaphyseal comminution)) treated with a below elbow CC or below elbow PS were included [13]. The decision for a CC or a PS was mostly based on the hospitals protocol and preference of the treating physician. Exclusion criteria comprised: no reduction performed, type of cast unknown, other types of immobilization than below elbow CC or PS, registered cast modifications during the first week of follow-up (e.g. cast cleavage), concomitant fracture of the ulna (except ulnar styloid process’ fractures), no radiographs available at one-week follow-up and failed reductions with unacceptable fracture displacement after reduction. Unacceptable fracture displacement is defined according to the Guidelines of the American Academy of Orthopaedic Surgeons [6]. This guideline defines displacement as radial shortening > 3 mm, or dorsal angulation > 10 degrees. General patient data (age, gender and fracture type) and treatment-related data (the type of cast and radiographs) were retrospectively reviewed.

### Outcomes

The primary outcome concerned the occurrence of fracture redisplacement at one-week follow-up, opting operative fixation as defined by the Guidelines of the American Academy of Orthopaedic Surgeons [6]. A subanalysis was performed for simple fractures and fractures with metaphyseal comminution. Furthermore, we analysed fracture angulation and radial length separately.

### Measurements

**Fig. 1:**
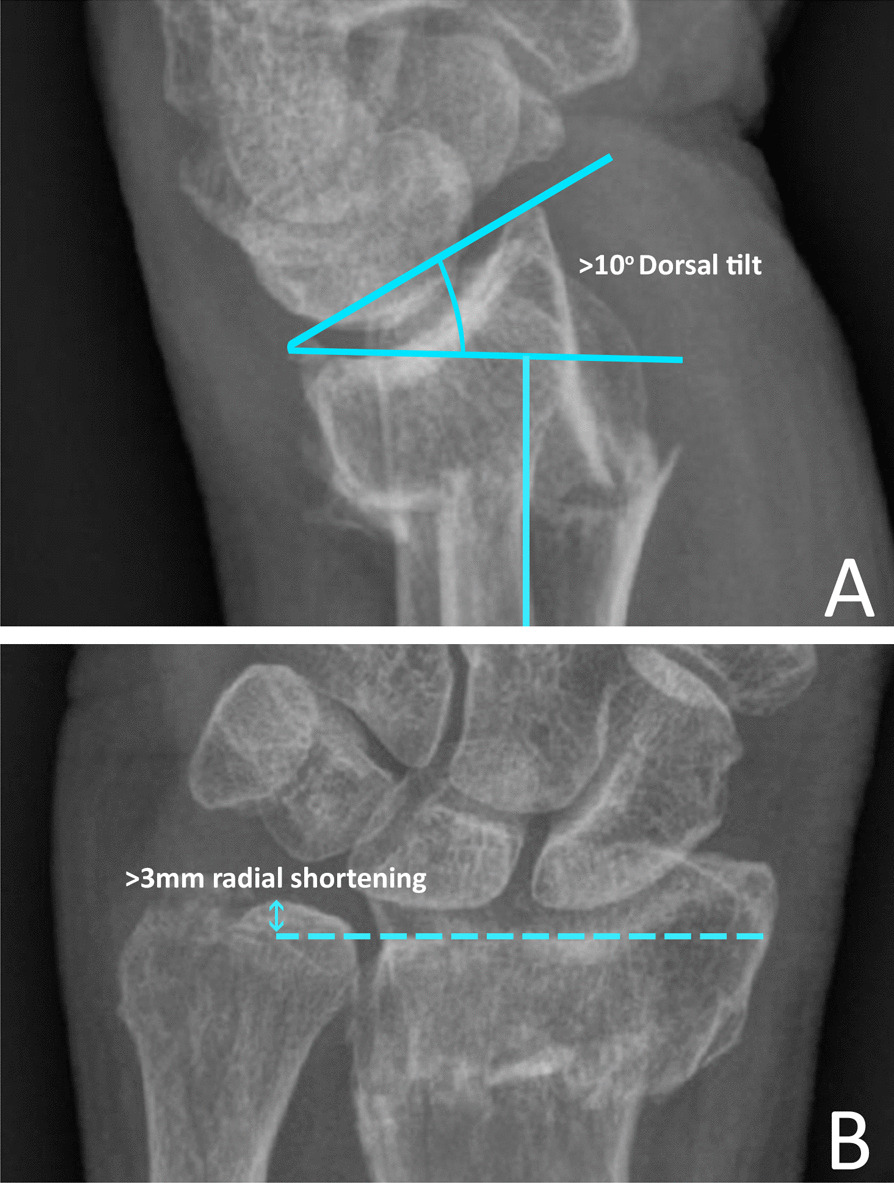
Fracture displacement measured according to the AAOS guideline. **A** The degree of angulation (volar or dorsal) was measured on the lateral view radiographs. This value represents the angle between a line along the distal radial articular surface and the line perpendicular to the longitudinal axis of the radius [10]. **B** Radial shortening was measured on the posteroanterior radiograph and refers to the distance between the carpal joint surface of the radius and the most distal part of the ulnar articular surface [14]

The degree of volar or dorsal angulation was measured on the lateral view radiographs. This value represents the angle between a line along the distal radial articular surface and a line perpendicular to the longitudinal axis of the radius (Fig. 1a) [10]. Radial shortening was measured on the posteroanterior radiograph and refers to the distance between the carpal joint surface of the radius and the most distal part of the ulnar articular surface (Fig. 1b) [14]. These measurements were taken 3 times: before reduction (T0), post-reduction (T1) and at 1-week follow-up (T2). Radiographic measurements were taken digitally in the locally available picture archiving and communication systems (PACS). To determine the direction of fracture redisplacement, the difference in angulation at T1 and T2 was used.

All measurements were taken by one researcher (AB). To evaluate inter- and intra-observer variability, 50 measurements were repeated and compared to one another by an orthopaedic surgeon (JC) and the researcher (AB).

### Statistical analysis

Statistical analyses were performed using SPSS (version 24; IBM). A *p* value of < 0.05 was considered significant. There were no missing data. Mann–Whitney *U* tests were used to describe baseline characteristics since they were all non-normally distributed. The Pearson Chi-square test was used to compare the appearance of fracture redisplacement after one-week follow-up between both groups. Differences in radial shortening and angulation were assessed using the independent samples Mann–Whitney *U* test. An intraclass correlation coefficient (ICC) was calculated to assess inter- and intra-observer variability in radiograph measurements. A two-way mixed-effects model was used, based on a single measurement with an absolute agreement definition. Values less than 0.5, between 0.5 and 0.75, between 0.75 and 0.9 and greater than 0.90 are indicative of poor, moderate, good and excellent, respectively [15].

## Results

### Patient selection

**Fig. 2:**
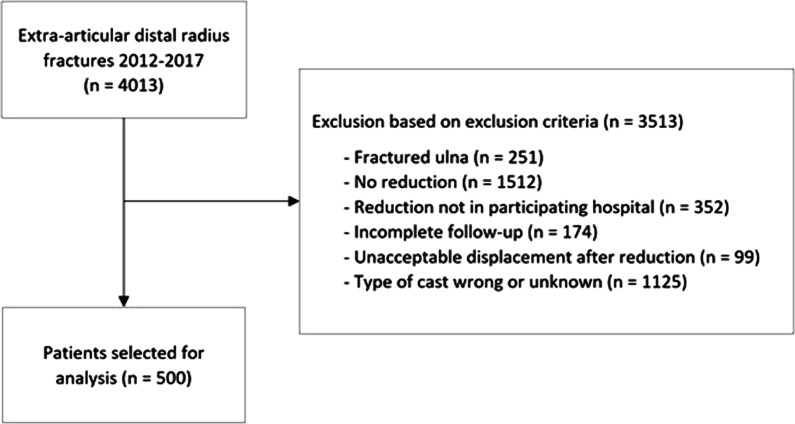
Flowchart of the selection process

The patient selection workflow is displayed in Fig. 2. The initial selection contained 4.013 patients treated in four hospitals. A total of 500 cases remained after eliminating patients meeting the exclusion criteria.

### Baseline characteristics

Baseline characteristics are displayed in Table 1. The PS group consisted of 184 patients and the CC group of 316 patients. Patients were predominantly female in both groups, namely 85% in the PS group and 91% in the CC group. The age distribution was similar in both groups. No between-group differences were observed concerning the severity of fracture displacement at admission. The PS group consisted of relatively more fractures with metaphyseal comminution (AO/OTA 23-A.3) compared to the CC group, respectively, 16% versus 9%. In both groups several DRFs were minimally displaced before reduction, meaning ≤ 3 mm radial shortening and ≤ 10 degrees of dorsal angulation. There was no significant difference concerning the distribution of minimally displaced fractures.

**Table 1:** Baseline characteristics

	Splint *n* = 184	Circumferential cast *n* = 316	*P* value
Female, *n* (%)	157 (85)	289 (92)	**0.033**
Age, years	66 (56; 79)	67 (56; 75)	0.738
Fracture displacement at T0			
Angulation*, degrees	21 (13; 27)	23 (14; 31)	0.055
Radial shortening, mm	− 2 (− 3; 0)	− 1 (− 3; 0)	0.622
Fracture classification (AO/OTA)			
Simple (AO/OTA 23-A.2), *n* (%)	154 (84)	289 (91)	**0.008**
Metaphyseal comminution (AO/OTA 23-A.3), *n* (%)	30 (16)	27 (9)	
Minimally displaced fractures^Ω^, *n* (%)	30 (16)	41 (13)	0.304

Statistically significant values are displayed in bold

Data are presented as medians with the interquartile range between parentheses

*n* = number of patients; T0 = at admission to emergency room

*Dorsal angulation is referred to as a positive number. In case of volar angulation, this is referred to as a negative number

^Ω^Minimally displaced fractures concern fractures with ≤ 3 mm radial shortening and ≤ 10 degrees of dorsal angulation

### Fracture displacement

At 1-week follow-up, fracture redisplacement occurred in 29% of patients treated with a PS compared to 17% in patients treated with a CC (*p* = 0.001). Similar results were found in subgroup analyses for simple fractures as well as fractures with metaphyseal comminution (*p* = 0.009 and *p* = 0.02, Table 2).

**Table 2:** Radiographic results

	Before reduction			At 1-week follow-up		
	Splint (*n* = 184)	Circumferential (*n* = 316)	*P* value	Splint (*n* = 184)	Circumferential (*n* = 316)	*P* value
Displaced fractures, *n* (%)	184	316	0.30	53	52	**0.001**
Simple fractures, n (%)	154	289	0.21	43	50	**0.009**
Fractures with metaphyseal comminution, *n* (%)	30	27	0.89	10	2	**0.023**

Statistically significant values are displayed in bold

*n* = number of patients

At 1-week follow-up, radial shortening occurred in 30% of fractures treated with a PS versus 15% of fractures treated with a CC (*p* = 0.038). Re-angulation was seen more often in fractures treated with a CC (75% vs 43%, *p* = 0.001). These results are displayed in Table 3.

**Table 3:** Radial shortening and angulation in redisplaced fractures at 1-week follow-up

Displaced fractures	Splint (*n* = 53)	Circumferential (*n* = 52)	*P* value
Radial shortening*, *n* (%)	16	7	**0.038**
Angulation^Ω^, *n* (%)	23	39	**0.001**
Radial shortening* and angulation^Ω^, *n* (%)	14	6	0.052

Statistically significant values are displayed in bold

*n* = number of patients

^*^Radial shortening > 3 mm

^Ω^Dorsal angulation > 10 degrees

### Inter- and intra-observer variability

intraclass correlation coefficients (ICCs) of 0.88 (95% CI 0.75–0.94) and 0.88 (95% CI 0.79–0.93) were determined for, respectively, inter- and intra-observer variability regarding the measurement of radial shortening. Regarding fracture angulation measurements ICCs of 0.67 (95% CI 0.49–0.80) and 0.66 (95% CI 0.47–0.79) were found for, respectively, inter- and intra-observer variability.

## Discussion

This study showed that one-week post-reduction, fracture redisplacement occurred almost twice as often in reduced DRFs immobilized with a below elbow PS compared to those treated with a below elbow CC (29% versus 17%). Amongst fractures with metaphyseal comminution (AO/OTA 23-A.3), almost five times as much redisplacement occurred in fractures treated with a PS compared to those treated with a CC (33% versus 7%).

A unique advantage of this study is the large number of patients included (*n* = 500). Patient selection took place in four hospitals, both academic and teaching hospitals, yielding a representative image of the patient population and treatment differences [16].

Concerning existing literature on this topic, only few articles focus on the influence of immobilization techniques on reduced DRFs. A systematic review by Handoll et al. (*n* = 4215) and more recent prospective studies by Grafstein et al. (*n* = 101), Wik et al. (*n* = 72) and O’Connor et al. (*n* = 66) compared circumferential casting (above- and below-elbow), plaster splints (dorsal and volar splints), synthetic splints (sugar-tong fibreglass splints, volar and dorsal fibreglass splints) and braces [9, 11, 17, 18]. In these studies, no significant differences were found between immobilization types regarding the occurrence of fracture redisplacement. Above-mentioned studies have different setups which make it hard to adequately compare outcomes. Grafstein et al. reported the loss of reduction in 16% of splinted DRFs versus 20% of circumferential casted DRFs [17]. However, in this study, loss of reduction was defined as the occurrence of radiologic slippage (based on radiographs of the complete casting period) or surgical fixation performed during the immobilization period. The definition of radiologic slippage is not further clarified in the article. O’Connor et al. performed a randomized controlled trial (*n* = 66) comparing a plaster cast with a lightweight removable splint [18]. In both groups, one patient suffered fracture displacement. Unfortunately, radiographic details and the type of plaster casting used, either splint or circumferential, are not mentioned.

Interestingly, loss of radial length occurred twice as much in fractures treated with splinting compared with fractures treated with circumferential casting. The outcome is conforming to previous research by Wik et al. [11]. Better preservation of radial length in reduced DRFs treated with a CC might be explained by more equal pressure distribution, both volarly and dorsally. This minimizes potential shearing and migration of the fracture. This theory is supported by a study of Alemdaroglu et al. They studied the impact of casting technique-related indices and found the three-point index to be useful in predicting fracture redisplacement (sensitivity of 96%, specificity of 96%) [3]. It makes sense to hypothesize that flattening a wet circumferential applied cast at the level of the wrist before it has hardened is essential to prevent redisplacement. Noteworthy to mention is that radial shortening seems to have the most significant negative impact on patient-reported outcomes during follow-up, making this parameter a potentially important factor in predicting outcome [19].

This study has its limitations. First, because of the retrospective design, patient-reported outcomes (e.g. pain scores, the comfort of casting) are not registered. However, multiple previous studies found no significant difference in pain severity when comparing circumferential casting to splinting [9, 11, 17, 18]. Second, we have no data available concerning the occurrence of adverse events. We consciously chose to focus on radiographic outcome alone. In our opinion, there is a high risk of bias searching for adverse events retrospectively. In particular, patient complaints and minor adverse events are not always reported consistently. A potential disadvantage of applying a CC directly after fracture reduction is the assumed higher risk of pressure-related problems, in ultimo reflected in a higher incidence of compartment syndrome of the forearm [20]. The occurrence of this serious complication is often used as an argument against the circumferential casting. However, the reported prevalence of compartment syndrome in unstable DRFs is very low (0–0.25%) and current knowledge of the prevalence in extra-articular DRFs is lacking [21, 22].

Third, we chose to include a select group of patients. Only extra-articular DRFs were included because the inter-observer variability of radiograph measurements in extra-articular fractures is lower compared to intra-articular fractures [23, 24]. We only included patients who did not encounter cast modifications during the first week of treatment to diminish the number of external factors that could possibly influence the reduction.

There was a difference regarding the distribution of extra-articular fractures with metaphyseal comminution. Relatively more fractures with metaphyseal comminution were found in the PS group. These fractures are considered to be more unstable compared to simple DRFs [7]. However, when excluding these fractures from the analysis, fracture redisplacement still occurred almost twice as much in the PS group.

Finally, this study focused on a limited timeframe of treatment, namely the first week. This point was chosen to minimize confounding by other factors that might influence the process of redisplacement (e.g. cast alterations or cast replacement). Thereby the included hospitals have different follow-up protocols which could influence the outcome. The results of this study should therefore be carefully interpreted as a first insight in the effect of immobilization on reduced DRFs.

## Conclusion

This study suggests that circumferential casting in reduced extra-articular distal radius fractures might cause less fracture redisplacement during the first treatment week compared to treatment with a plaster splint. Fracture redisplacement occurred twice as much in patients treated with a plaster splint compared to treatment with a circumferential cast. Important questions about functional outcome, complication risks and patient-reported outcomes are still to be answered. Therefore, a randomized controlled trial will be conducted to confirm the current findings, taking functional outcome, complication risks and patient-reported outcome into account [25].

## Data Availability

The datasets used and/or analysed during the current study are available from the corresponding author on reasonable request.

## Abbreviations

DRFs: Distal radius fractures
PS: Plaster splint
CC: Circumferential cast
